# Editorial: Cancer cell-intrinsic and -extrinsic factors affecting tumor immune evasion

**DOI:** 10.3389/fimmu.2023.1261820

**Published:** 2023-07-31

**Authors:** Jiazheng Sun, Andrey A. Zamyatnin, Xiangliang Yuan, Yi Xiao, Hongzhong Li

**Affiliations:** ^1^ Chongqing Key Laboratory of Molecular Oncology and Epigenetics, The First Affiliated Hospital of Chongqing Medical University, Chongqing, China; ^2^ Scientific Center for Genetics and Life Sciences, Division of Biotechnology, Sirius University of Science and Technology, Sochi, Russia; ^3^ Institute of Molecular Medicine, Sechenov First Moscow State Medical University, Moscow, Russia; ^4^ Belozersky Institute of Physico-Chemical Biology, Lomonosov Moscow State University, Moscow, Russia; ^5^ Faculty of Health and Medical Sciences, University of Surrey, Guildford, United Kingdom; ^6^ Department of Molecular and Cellular Oncology, The University of Texas MD Anderson Cancer Center, Houston, TX, United States

**Keywords:** immune checkpoint, tumor microenvironment, suppressive immune cell, tumor heterogeneity, immune evasion

## Introduction

Multiple types of human tumors employ mechanisms to suppress the immune system and enhance their own survival. One such method is the evasion of immune recognition by tumor cells through the reduction of specific antigen-presenting proteins on their surface. This renders these cells invisible to cytotoxic T lymphocytes (1). Tumor cells also secrete proteins that suppress the response of effector T cells and promote the proliferation and functionality of immunosuppressive cells that actively hinder immune responses. Additionally, cancer cell-extrinsic factors, such as myeloid-derived suppressor cells (MDSCs), tumor-associated macrophages (TAMs), regulatory T cells (Tregs), serve as significant components of the immune suppressive tumor microenvironment (TME). These factors contribute to the dysfunction of T-cells, ultimately facilitating tumor progression (2).

This Research Topic encompasses a collection of reviews, opinions, perspectives, and primary research articles that investigate the influence of cancer cell-intrinsic and -extrinsic factors on tumor immune evasion.

## Exploration of the unknown in cancer cell-intrinsic and -extrinsic factors affecting tumor immune evasion

Despite significant research on cancer cell-intrinsic and -extrinsic factors, there are still several aspects that require further investigation. The scientific researchers contributing to this Research Topic have presented intriguing research directions that pave the way for future possibilities in the field.

Cancer cell-intrinsic factors, such as genomic instability, epigenetic alterations, altered cancer cell metabolism, and oncogenic signaling, can be associated with resistance to immune checkpoint blockade. Ursino et al.‘s review discuss the association between these factors and the response to immunotherapy.

Tumors with deficient mismatch repair (MMR) protein expression are characterized by an inflamed tumor microenvironment (TME), which plays a crucial role in the clinical efficacy of anti-PD-1 therapy for patients with this condition. However, a significant proportion (ranging from 40% to 70%) of cancer patients with deficient MMR do not respond to PD-1 blockade treatment. Mestrallet et al. summarize recent discoveries regarding the mechanisms of immune evasion in MMR-deficient (MMRd) tumors during checkpoint blockade treatment.

Glioblastoma is one of the most aggressive malignancies in the central nervous system, characterized by strong invasion, frequent recurrence, and rapid progression. These features are closely associated with immune evasion, presenting a significant obstacle to glioma treatment. The lysosomal peptidase family plays a crucial role in the immune evasion of glioma. Liu et al.‘s review specifically focus on the regulatory effects of lysosomal peptidases on immune evasion and their underlying mechanisms.

About 80% of pancreatic cancers are detected at an advanced stage, making them challenging to treat. While complex surgical operations are one of the most effective approaches, the rate of radical resection is less than 20%. Additionally, pancreatic cancer exhibits significant resistance to chemotherapy and radiotherapy. Pancreatic cancer is known to establish an immune-suppressive microenvironment that evades the host antitumor immune system, contributing to rapid cancer progression. Therefore, immunotherapy has garnered significant expectations and become a crucial treatment alongside chemoradiotherapy. Li et al. summarize the mechanisms of stromal cells during the formation of the immunosuppressive TME, as well as the potential clinical application of immunotherapy strategies.

Endometrial carcinoma (EC) is the most common malignant tumor of the female reproductive system. Although surgery can cure the majority of EC patients, those with more aggressive variants still face a poor prognosis. The spatial heterogeneity of tumor-infiltrating lymphocytes (TILs) is related to the prognosis of EC patients. Yu et al. used single-cell and spatial transcriptomic analyses to explore the heterogeneity of EC. They revealed that EC cells could shape the TME by suppressing immune cells and “educating” stromal cells through the MDK-NCL signaling pathway.

ERIANIN is a natural biphenyl compound extracted from biphenyl compound, and its tumor-suppressive effects have been validated in numerous studies involving various diseases. Yang et al.‘s review summarize the studies of ERIANIN in cancer and innate immunity, and explain its molecular mechanism associated with anticancer activity through several signaling pathways including the MEK pathway, PI3K/AKT pathway, JNK pathway, NRF2/PLOOH pathway, JAK/STAT3 pathway, GSK3β pathway, and NLRP3/ROS pathway.

## Detection of tumor predictive marker and Potential therapeutic target

Phosphoinositide 3-kinases (PI3Ks) are a family of lipid enzymes involved in various intracellular functions. PIK3C2A, a classical member of the PI3K class II, is implicated in numerous cancer-related pathways. Qin et al. demonstrate that PIK3C2A is an independent biomarker for tumor progression and regulates immune infiltration in several malignancies. In the case of kidney renal clear cell carcinoma (KIRC), elevated expression of PIK3C2A is associated with a more favorable prognosis and increased infiltration of immune effector cells. Moreover, aberrant expression of PIK3C2A can modulate the proliferative capacities of KIRC.

CD147, a type of glycoprotein, plays a significant role in tumor cell invasion, metastasis, and angiogenesis. Huang et al. describe the molecular structure of CD147 and its crucial involvement in regulating the invasion, metastasis, and angiogenesis of hepatocellular carcinoma (HCC).

Soluble PD-L1 (sPD-L1) is an important form of PD-L1, detectable in the serum of cancer patients with certain viral diseases or autoimmune diseases. sPD-L1 has been identified in more than 20 distinct pathological conditions and often plays a significant immunoregulatory role. In the context of cancer, sPD-L1 has been detected in plasma, and its increased levels have been associated with poor prognosis in patients. Liang et al. discovered that soluble PD-L1 is capable of inhibiting the function of activated T cells and promoting peripheral tolerance to tumor cells.

## Conclusion

The Research Topic titled “*Cancer Cell-intrinsic and -extrinsic Factors Affecting Tumor Immune Evasion*” has compiled several notable studies and valuable contributions related to tumor immune evasion. These contributions provide valuable insights into novel directions for researching tumor immune escape. The complex interactions among suppressive immune cells, immunoregulatory cytokines or signaling, and cancer cell-intrinsic factors establish a permissive tumor microenvironment (TME) that facilitates immune evasion and promotes tumor growth. In summary, the Research Topic highlights the crucial role of cancer cell-intrinsic and -extrinsic factors in immune evasion and their association with the effectiveness of cancer immunotherapy. We remain optimistic that these efforts will contribute to overcoming immunotherapy resistance and enhancing its efficacy.

## Author contributions

HL: Writing – review & editing. JS: Writing – original draft. AZ: Writing – review & editing. XY: Writing – review & editing. YX: Writing – review & editing.
